# Insomnia Symptoms in Patients With Substance Use Disorders During Detoxification and Associated Clinical Features

**DOI:** 10.3389/fpsyt.2020.540022

**Published:** 2020-11-17

**Authors:** Lara Grau-López, Laia Grau-López, Constanza Daigre, Raúl Felipe Palma-Álvarez, Nieves Martínez-Luna, Elena Ros-Cucurull, Jose Antonio Ramos-Quiroga, Carlos Roncero

**Affiliations:** ^1^Department of Psychiatry, Hospital Universitari Vall d'Hebron, Barcelona, Spain; ^2^Group of Psychiatry, Mental Health and Addiction, Vall d'Hebron Research Institute, Barcelona, Spain; ^3^Biomedical Network Research Centre on Mental Health (CIBERSAM), Barcelona, Spain; ^4^Department of Psychiatry and Forensic Medicine, Autonomous University of Barcelona, Barcelona, Spain; ^5^Department of Neurosciences, Hospital Universitari Germans Trias i Pujol, Badalona, Spain; ^6^Psychiatric Service, University of Salamanca Health Care Complex, Institute of Biomedicine of Salamanca, University of Salamanca, Salamanca, Spain; ^7^Psychiatric Unit, School of Medicine University of Salamanca, Salamanca, Spain

**Keywords:** addiction, insomnia, sleep disorders, women, detoxification process, anxiety disorders

## Abstract

**Background:** Insomnia is highly prevalent in patients with substance use disorders (SUD), and it has been related to a worse course of addiction. Insomnia during detoxification in a hospital has not been adequately studied. This study aims to compare sociodemographic, clinical, and psychopathological characteristics of SUD patients undergoing a detoxification program, by comorbidity and insomnia symptoms.

**Methodology:** We recruited 481 patients who received pharmacological and psychotherapeutic treatment for detoxification. They were evaluated through semi-structured interviews, standardized questionnaires, and a specific sleep log. A bivariate and multivariate analysis of the data was performed.

**Results:** Insomnia was reported by 66.5% patients, with sleep-maintenance insomnia the most frequent issue, followed by early morning awakening and sleep-onset insomnia. Patients with alcohol use disorder and cannabis use disorder had higher prevalence of sleep-onset insomnia. Patients with cocaine and heroin use disorder had higher prevalence of sleep-maintenance insomnia. Independent factors that allowed the identification of insomnia symptoms included being female (OR: 3.43), polysubstance use (OR: 2.85), comorbid anxiety disorder (OR: 2.02), and prior admission for detoxification (OR: 1.22).

**Conclusions:** Insomnia symptoms are very prevalent in patients admitted for detoxification. The diagnosis and therapeutic strategies for the insomnia symptoms should be improved, especially in women and in patients with greater addiction severity and with anxiety disorders.

## Introduction

Insomnia has been defined as a sleep disorder characterized by difficulty with sleep initiation, duration, consolidation, or quality that occurs despite circumstances favorable for sleeping. In addition, there could be daytime impairment symptoms, including fatigue, sleepiness, mood disturbances, physical symptoms and concerns about or dissatisfaction with sleep (1).

Insomnia is estimated to affect between 20 and 35% of the general population (2–6) and 40–90% of patients with psychiatric disorders other than addictions (7–10). Individuals with substance use disorders (SUD) also have a higher prevalence of insomnia than the general population (11, 12). The relationship is bidirectional; people with insomnia are also more prone to SUD (13–15). Insomnia is suffered by between 30 and 85% of SUD patients, depending on the primary substance of abuse and other methodological factors (16–19). Alcohol use disorder is the problem most studied regarding insomnia symptoms, and the rates described for those patients are between 30 and 60% (20–22). Sleep in this population is influenced by numerous substance use-related factors, such as type and amount of drug used, duration of addiction, route of administration, state of intoxication or withdrawal, and simultaneous consumption of stimulants and depressants.

In the general population, insomnia is more likely to affect women, people over 65 years old, and shift and night workers (23–25). Likewise, patients with medical and psychiatric comorbidities are more susceptible to nocturnal sleep disturbances (4, 7).

Insomnia has been linked to a worsening of addiction (26, 27); however, few studies have analyzed risk factors for insomnia in patients with SUD. It has been identified that comorbid anxiety disorder, medical comorbidities, and early onset of dependence increase the risk of insomnia during active substance use (28).

Detoxification is frequently required among SUD patients, who could be done in an outpatient or hospital setting. The detoxification process is a critical time to evaluate and propose therapeutic approaches, including insomnia symptoms. It has been found that symptoms of insomnia during a detoxification process are a risk factor to early relapses (26). To our knowledge, however, risk factors of insomnia symptoms during detoxification process have not been described. Therefore, this study aimed to compare sociodemographic, clinical, and psychopathological characteristics of SUD patients undergoing a detoxification program according to the presence/absence of insomnia symptoms. It was hypothesized that symptoms of insomnia would be common in this population and that patients with insomnia symptoms would have higher rates of psychiatric comorbidity and more severe addiction.

## Methods and Materials

### Participants

In this study 481 SUD inpatients participated in the detoxification unit of Vall d'Hebron University Hospital in Barcelona, Spain. The recruitment period was from June 2013 to May 2018.

The inclusion criteria were substance dependence (excluding primary abuse of caffeine or nicotine) in the past year according to DSM criteria, active consumption up to the day before admission to the detoxification unit, and provision of signed informed consent to participate in the study. The exclusion criteria were failure to complete the detoxification program and presence of a major language barrier or severe cognitive impairment. The study was approved by the Vall d'Hebron Hospital ethics committee, according to the (29).

### Procedure

Patients received appropriate detoxification treatment for their SUD. The treatment in all cases consisted of pharmacologic and psychological interventions to treat SUD (detoxification) and psychiatry comorbidity. They received decreasing doses of benzodiazepines (regardless of the substance that led to admission) and adjuvant medications to treat comorbid psychiatric symptoms. Patients participated in group psychotherapy during the detoxification program, which consisted of three sessions including psychoeducation, a motivation interview, and prevention of substance addiction relapse. Hospital stays ranged between 10 and 15 days (10.9 ± 3.2).

The assessment process consisted of an evaluation of insomnia symptoms conducted by daily interviews with a psychiatrist and through information from sleep logs kept by night-shift nurses. In addition, three interviews conducted by psychologists following the protocol to evaluate comorbidity were done.

### Study Variables and Instruments

The variables recorded were insomnia symptoms, sociodemographics, substance consumption variables, and medical and psychiatric comorbidities.

**Figure 1:**
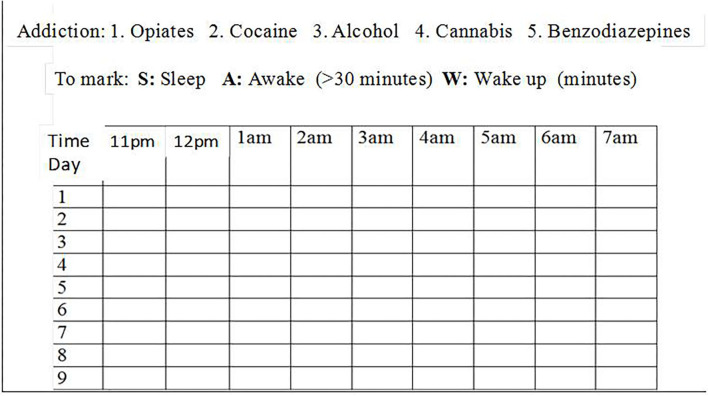
Nighttime sleep log. S, sleep; A, awake (>30 min); W, wake up (minutes).

The presence of symptoms of insomnia was evaluated by the psychiatrist according to clinical criteria, based on daily interviews and complementary information from a sleep log. The sleep log is a tool where the night-shift nurses recorded how each patient slept for each hour between 11 p.m. and 7 a.m. was considered in order to diagnose insomnia symptoms (Figure 1). A distinction was made between the following types of insomnia: sleep-onset insomnia (failure to fall asleep within the first half hour of going to bed), sleep-maintenance insomnia (two or more awakenings a night), early awakening (awakening at least 1 h before the usual time), poor sleep quality (none of the above but presence of diurnal effects, such as daytime sleepiness, fatigue, irritability, nervousness, decreased concentration, muscle tension, and headache).

Patients who were diagnosed with insomnia symptoms were prescribed specific treatment according to their profile and the judgment of the clinicians responsible for their care.

Psychiatric comorbidity was evaluated using semi-structural diagnostic interviews. SCID-I (Structured Clinical Interview for the DSM-IV Axis I Disorders), SCID-II (Structured Clinical Interview for the DSM-IV Axis II Disorders), and CAADID (Conners' Adult ADHD Diagnostic Interview for DSM-IV) were used to diagnose attention deficit hyperactivity disorder (ADHD). Urinalysis and alcohol breath test results were used to evaluate substance consumption at the first day of the detoxification process.

### Statistical Analysis

First of all, the main variables of the sample were analyzed using descriptive statistics (mean, standard deviation, and frequency tables) and bivariate analyses. Later, in order to conduct comparisons, the sample was divided into two groups (patients with insomnia symptoms vs. patients without insomnia symptoms). The chi-square test was used to compare categorical variables between these groups, while Student's *t*-test was used to compare continuous variables. Afterward, to reduce false positive results, a Bonferroni correction for multiple tests was applied according to the number of tests in each bivariate analysis. Variables that retained statistical significance after Bonferroni correction were included in the multivariate analysis. Finally, logistic regression, with the enter method, was used to identify the independent effect of the variables of the insomnia symptoms (present of insomnia = 1). Data were entered and analyzed in SPSS version 18.0. In all cases, statistical significance was set at *p* < 0.05.

## Results

### Description of the Sample

A total of 552 patients were admitted to the detoxification unit during the study period. Fifty patients requested voluntary discharge before completion of the program, and 21 were not included because of language barriers or severe cognitive impairment. Hence, the final sample consisted of 481 patients.

Table 1 summarizes the sociodemographic, clinical, and treatment characteristics of the sample. The most common primary substance of use was alcohol (42.4%), followed by cocaine (32.8%), opiates (12.9%), cannabis (7.1%), and benzodiazepines (4.8%). Polysubstance dependence (three substance use disorders within the past 12 months) was reported for 46.4% patients. Polysubstance dependence (three substance use disorders within the past 12 months) was reported for 46.4% patients. The distribution of substance consumption across the lifespan of the polysubstance dependent patients was 85.8% cocaine, 77.7% alcohol, 75.2% cannabis, 56.7% opioids, and 54.1% benzodiazepines.

**Table 1:** Description of study participants.

**Sociodemographic variables**	**Total (*n* = 481)**		**Total (*n* = 481)**
Age (years)	40.9 ± 10.3	Employed (working)	16.2%
Gender (male)	72.3%	Marital status (married)	33.1%
Nationality (foreign)	9.8%	Living with someone	34.1%
Education (primary education)	66.7%	Criminal record (prison)	20.6%
**Clinical variables related to comorbidities**			
Medical history	60.7%	Axis II disorders	44.7%
Psychiatric history	62.2%	Cluster B	28.7%
Axis I disorders	43.7%	Other personality disorder	9.3%
Anxiety disorder	15.6%	Cluster C	4.8%
Depressive disorder	14.1%	Cluster A	1.9%
Attention deficit hyperactivity disorder	13.1%		
Psychotic disorder	8.5%	Prior psychiatric admissions	15.6%
Bipolar disorder	3.1%		
**Clinical variables related to consumption**			
Age at start of consumption	17.8 ± 6.4	Polysubstance users	46.4%
Age at onset of substance use disorder	24.1 ± 8.2	Binge consumption prior to admission	55.3%
Years of addiction	16.6 ± 11.3	Positive breath alcohol test on admission	25.2%
Main Substance Use Disorder		Positive urinalysis on admission	55.9%
Alcohol	42.4%	Prior detoxification admissions	52%
Cocaine	32.8%		
Heroine	12.9%		
Cannabis	7.1%		
Benzodiazepines	4.8%		
**Psychopharmacological and Psychological treatment**			
Antiepileptic	69%	ADHD drugs	4.7%
Antidepressant	60.1%	Opioid agonists	12.9%
Antipsychotic	44.9%	Disulfiram/Namelfeno	20.8%
Benzodiazepines	6.9%	Group Psychotherapy	81.1%
**Insomnia symptoms**			
No insomnia symptoms during detoxification process	33.5%	Type of insomnia	
		Sleep-maintenance insomnia	45.1%
		Early morning awakening	25.4%
Insomnia symptoms during detoxification process	66.5%	Sleep-onset insomnia	16.4%
		Poor quality of nocturnal sleep	12.3%

Insomnia symptoms were reported by 66.5% of patients (*n* = 320) during the detoxification process and 84.3% reported previous insomnia symptoms. Sleep-maintenance insomnia was the most common type of insomnia overall, followed by early morning awakening, sleep-onset insomnia, and poor quality of nocturnal sleep. At discharge, insomnia symptoms were considered to have been alleviated in 63.8% of patients

On the other hand, the relationship among type of insomnia symptoms and main SUD was analyzed. Patients with alcohol use disorder had higher prevalence of sleep-onset insomnia (44.7 vs. 30.8%, *p* < 0.02), early awakening (45.4 vs. 33.6%, *p* < 0.02) and poor sleep quality (44.2 vs. 11.5%, *p* < 0.001) than other SUD patients. Patients with cocaine use disorder had higher rates of sleep-maintenance insomnia (37.5 vs. 27.2%, *p* < 0.02) and poor sleep quality (57.7 vs. 31.4%, *p* < 0.006). Patients with heroin use disorder were more likely to experience sleep-maintenance insomnia (21.2 vs. 6.1%, *p* < 0.0001). Finally, patients with cannabis use disorder had higher prevalence of sleep-onset insomnia (14.1 vs. 5.7%, *p* < 0.008), while patients with benzodiazepine use disorder had higher prevalence of sleep-maintenance insomnia (8.3 vs. 1.9%, *p* < 0.001) and early awakening (8.2 vs. 3.6%, *p* < 0.04) than patients with other SUD.

### Insomnia Symptoms According to Sociodemographic and Clinical Variables

As is shown in Table 2, insomnia symptoms were more frequently present in women and older individuals. Patients with heroin use disorders had significantly more insomnia symptoms than patients with another substance use disorders. Also, polysubstance users and patients with medical conditions had significantly more insomnia symptoms. When comorbidity psychiatry was compared for insomnia symptoms, that anxiety disorders, ADHD, cluster B personality disorder, and prior admissions for detoxification more were found frequently (see Table 2).

**Table 2:** Insomnia symptoms according to sociodemographic and clinical variables during detoxification process.

**Sociodemographic variables**	**Insomnia symptoms (*n* = 320)**	**No insomnia symptoms (*n* = 161)**	***p***
Age (years)	44.4 ± 11.8	40.2 ± 9.3	0.04^*^
Gender (female)	34.7	13.7	0.0001^*^, ^**^
Nationality (foreign)	9.1	11.2	0.46
Education (primary education)	32.5	34.8	0.62
Employed (working)	88.3	86.6	0.52
Marital status (married)	30.3	38.5	0.07
Living with someone	30.6	31	0.21
Criminal record (prison)	24.9	23.4	0.78
**Clinical variables related to comorbidities**			
Medical history	64.7	52.8	0.01^*^
Psychiatric history	68.1	60.3	0.10
Axis I	49.4	42.3	0.18
Anxiety disorder	19.4	8.1	0.001^*^
Psychiatric history	68.1	60.3	0.10
Axis I	49.4	42.3	0.18
Anxiety disorder	19.4	8.1	0.001^*^, ^**^
Depressive disorder	19.6	16.6	0.32
Attention deficit hyperactivity disorder	15.3	8.7	0.04^*^
Psychotic disorder	8.4	8.7	0.92
Bipolar disorder	3.8	1.9	0.26
Axis II	51.6	43.1	0.09
Cluster B	33.1	19.9	0.002^*^, ^**^
Other personality disorder	11.6	10.1	0.81
Cluster C	5	4.3	0.75
Cluster A	1.9	1.9	0.99
Prior psychiatric admissions	26.8	20	0.09
**Clinical variables related to consumption**			
Age at start of consumption	17.9 ± 6.4	17.5 ± 6.4	0.44
Age of onset of substance use disorder	24.3 ± 8.04	23.8 ± 8.6	0.55
Years of addiction	19.6 ± 10.2	18.6 ± 13.0	0.87
Main Substance Use Disorder	%	%	
Alcohol	57.8	51.6	0.40
Cocaine	31.9	34.8	0.52
Heroine	16.6	5.6	0.001^*^, ^**^
Cannabis	6.6	8.1	0.54
Benzodiazepines	7.2	5.2	0.11
Polysubstance users	56.6	26.1	0.0001^*^, ^**^
Prior detoxification admissions	59.7	36.6	0.0001^*^, ^**^
Binge consumption prior to admission	53.3	53.3	0.99
Positive breath alcohol test	23.8	28	0.32
Positive urinalysis	61.3	55.3	0.08
**Psychopharmacological and psychological treatment**			
Antiepileptic	71.3	82.2	0.45
Antidepressant	59.3	76.3	0.38
Antipsychotic	45.3	57.2	0.21
Benzodiazepines	6.9	6.8	0.986
Opioid agonists	9.3	16.3	0.08
Interdictors	17.9	23.8	0.73

* *p < 0.05*;

** *Statistically significant after Bonferroni correction*.

The following variables were included in the logistic regression analysis after Bonferroni correction: gender (female), polysubstance users, anxiety disorder, prior admission for detoxification, admission for heroin detoxification, and cluster B personality disorder. Independent factors that allowed for identification of insomnia symptoms were female, polysubstance use, prior admission for detoxification, and comorbid anxiety disorder. Women were 3.43 times more likely to suffer from insomnia. Patients with a history of polysubstance use were 2.85 times more likely more likely to suffer from insomnia, and patients with anxiety disorders were 2.02 times more likely to suffer from insomnia. Finally, patients with history of prior admission for detoxification were 1.22 times more likely to suffer from insomnia during the detoxification process (see Table 3).

**Table 3:** Variables independently associated with insomnia symptoms during detoxification process.

	**OR**	**95% CI**	***p***
Gender (female)	3.43	2.01–5.86	0.0001
Polysubstance users	2.85	1.79–4.54	0.0001
Anxiety disorder comorbidity	2.09	1.06–4.12	0.03
Prior admission for detoxification	1.22	1.07–1.38	0.002
Admission for heroin detoxification	1.77	0.80–3.94	0.16
Cluster B personality disorder	1.22	0.74–2.03	0.44

## Discussion

We detected a high prevalence of insomnia symptoms (66.5%) in the early stages of substance withdrawal in patients admitted to a hospital detoxification unit. This rate is higher than the rates of 20–35% reported for the general population (2–6) and similar to rates described for patients with other psychiatric disorders (7–10, 30–32). In a previous study, we found that 84.3% of addicted patients experienced insomnia symptoms during active consumption of substances (28). It could be hypothesized, that patients actively consuming such substances have less frequent insomnia symptoms because hypersomnia is an expected withdrawal symptom of the stimulant detoxification process.

The prevalence of insomnia symptoms is high, and it ranges from 59.3 to 100%, depending on the primary substance used. These rates are consistent with insomnia prevalence described in other studies on SUD (17–22). Behavioral and psychobiological factors increase the prevalence of insomnia symptoms in SUD patients, for example, bad sleep hygiene and the interaction between the hypocretinergic and dopaminergic systems (13). As for the type of insomnia, sleep-maintenance insomnia was observed in half of the patients and was the most common type of insomnia identified. A similar rate has been reported in other substance-use disorder patients (13).

In the multivariate analysis, the independent risk factors associated with insomnia symptoms were female sex, anxiety disorder comorbidity, polysubstance use and prior admission for detoxification.

We found that women in our study have more insomnia symptoms, just as they do in the general population. According to diverse studies and revisions, insomnia in women might be increased and affected by hormonal changes such as menstruation, pregnancy, breastfeeding, or menopause (23). Another explanation is that women tend to suffer anxiety more frequently, as well as depressive disorders associated with insomnia (23). In the same way, it has been found that anxiety disorders are independently associated with insomnia symptoms in SUD patients. The relationship between insomnia and anxiety disorders is unknown; however, at the neurobiological level it has been proposed that the excitability of the right parietal cortex in patients with generalized anxiety disorder comorbid with insomnia was modulated by insomnia (33). Moreover, the inclusion of insomnia symptoms among the diagnostic criteria of anxiety and depressive disorders increases the probability for these comorbidities (34).

Polysubstance use understood as a history of addiction to three or more substances was identified as an independent risk factor for insomnia symptoms. The interaction between different withdrawal symptoms could partially explain this association. Patients who had been previously admitted for detoxification were also more likely to experience insomnia symptoms. These two factors can be considered as indicators of addiction severity that are closely associated with insomnia symptoms; therefore, insomnia could be a frequent symptom in patients with worse addiction treatment outcomes. In line with this, it is coherent that insomnia has been associated with early relapses in both alcohol use disorders and illegal substance use disorders patients (26, 27). It is important to treat insomnia symptoms because they have been identified as a risk factor for relapses after a detoxification process (26). A recent study has suggested that SUD patients who have been pharmacologically treated and have improved their insomnia symptoms maintained abstinence from substance use for longer in comparison with patients who did not improve from their insomnia symptoms (35). These findings highlight the need to treat insomnia symptoms during the therapeutic process.

Other significant factors in the bivariate analysis were age, ADHD, Cluster B personality, medical comorbidities, and heroin addiction. All these features have been identified as risk factors for insomnia in previous studies (3, 4, 7). Thus, psychiatry and medical comorbidities should be treated in order to improve insomnia symptoms and the quality of life of SUD patients (36).

Certain limitations of this study should be considered. First, electrophysiological tests such as polysomnography and actigraphy were not used to assess insomnia. However, it has been established that these objective measures should not take preference over clinical evaluation and clinical judgment. On the other hand, it should be considered that participants of this study were enrolled in a detoxification program and probably they were heavier users than patients seen in outpatient drug clinics. However, most patients with an SUD need to undergo detoxification at different stages of their addiction. It should also be noted that medications prescribed to patients during hospital detoxification may influence the presence or absence of insomnia symptoms, although all the patients in our study underwent a similar psychopharmacology approach. Unfortunately, the severity of insomnia was not included in the design; however, our future studies will include it.

Our study also has several strengths. The fact that we used psychometric tests to analyze a large sample of patients lends strength to the results, providing validity to the clinical diagnosis and the presence of insomnia symptoms. Finally, our findings also add to the limited data on the prevalence of symptoms of insomnia in patients with substance use disorders other than alcohol (12, 28).

We confirm that insomnia symptoms are very common among SUD patients during detoxification, especially the subtype sleep-maintenance insomnia (13). Polydrug use, comorbid anxiety disorder, prior admission to detoxification programs, and being female were independent factors associated with insomnia symptoms. A specific assessment and more psychotherapeutic and pharmacological strategies are needed that consider the symptoms of insomnia within the integral treatment of addictions, which is a common concern among these patients and a risk factor for relapse (12, 14, 27, 28). Sleep hygiene interventions should be included in the management of SUD patients in order to improve their quality of life and course of addiction treatment.

## Data Availability Statement

The datasets generated for this study are available on request to the corresponding author.

## Ethics Statement

The studies involving human participants were reviewed and approved by Psychiatry Department, Vall d' Hebron University Hospital. The patients/participants provided their written informed consent to participate in this study.

## Author Contributions

All authors listed have made a substantial, direct and intellectual contribution to the work, and approved it for publication.

## Conflict of Interest

The authors declare that the research was conducted in the absence of any commercial or financial relationships that could be construed as a potential conflict of interest.

## References

[1] The International Classification of Sleep Disorders. Diagnostic and Coding Manual. 3rd ed Hanover: American Academy of Sleep Medicine (2014).

[2] Buysse DJ Insomnia. JAMA. (2013) 309:706–16. 10.1001/jama.2013.193 23423416 PMC3632369

[3] Chung KF Yeung WF Ho FY Yung KP Yu YM Kwok CW. Cross-cultural and comparative epidemiology of insomnia: the Diagnostic and statistical manual (DSM), International classification of diseases (ICD) and International classification of sleep disorders (ICSD). Sleep Med. (2015) 16:477–82. 10.1016/j.sleep.2014.10.018 25761665

[4] Baglioni C Nanovska S Regen W Spiegelhalder K Feige B Nissen C. Sleep and mental disorders: a meta-analysis of polysomnographic research. Psychol Bull. (2016) 142:969–90. 10.1037/bul0000053 27416139 PMC5110386

[5] Hamblin JE. Insomnia: an ignored health problem. Prim Care. (2007) 34:659–74. 10.1016/j.pop.2007.05.009 17868765

[6] Cunnington D Junge MF Fernando AT. Insomnia: prevalence, consequences and effective treatment. Med J Aust. (2013) 199:36–40. 10.5694/mja13.10718 24138364

[7] Sutton EL. Psychiatric disorders and sleep issues. Med Clin North Am. (2014) 98:1123–43. 10.1016/j.mcna.2014.06.009 25134876

[8] Khurshid KA. Comorbid insomnia and psychiatric disorders: an update. Innov Clin Neurosci. (2018) 15:28–32. 29707424 PMC5906087

[9] Abad VC Guilleminault C. Sleep and psychiatry. Dialogues Clin Neurosci. (2005) 7:291–303. 16416705 10.31887/DCNS.2005.7.4/vabad PMC3181745

[10] Spoormaker VI Van den Bout J. Depression and anxiety complaints; relations with sleep disturbances. Eur Psychiatry. (2005) 20:243–5. 10.1016/j.eurpsy.2004.11.006 15935423

[11] Cheng SH Shih CC Lee IH Hou YW Chen KC Chen KT. A study on the sleep quality of incoming university students. Psychiatry Res. (2012) 197:270–4. 10.1016/j.psychres.2011.08.011 22342120

[12] Roncero C Grau-López L Díaz-Morán S Miquel L Martínez-Luna N Casas M. [Evaluation of sleep disorders in drug dependent inpatients]. Med Clin. (2012) 138:332–5. 10.1016/j.medcli.2011.07.015 22018396

[13] Cañellas F de Lecea L. [Relationships between sleep and addiction]. Adicciones. (2012) 24:287–90. 10.20882/adicciones.78 23241715 PMC5047372

[14] Roth T. Does effective management of sleep disorders reduce substance dependence? Drugs. (2009) 69:65–75. 10.2165/11531120-000000000-00000 20047351

[15] Teplin D Raz B Daiter J Varenbut M Tyrrell M. Screening for substance use patterns among patients referred for a variety of sleep complaints. Am J Drug Alcohol Abuse. (2006) 32:111–20. 10.1080/00952990500328695 16450646

[16] Dimsdale JE Norman D DeJardin D Wallace MS. The effect of opioids on sleep architecture. J Clin Sleep Med. (2007) 3:33–6. 17557450

[17] Babson KA Sottile J Morabito D. Cannabis, cannabinoids, and sleep: a review of the literature. Curr Psychiatry Rep. (2017) 19:23. 10.1007/s11920-017-0775-9 28349316

[18] Schierenbeck T Riemann D Berger M Hornyak M. Effect of illicit recreational drugs upon sleep: cocaine, ecstasy and marijuana. Sleep Med Rev. (2008) 12:381–9. 10.1016/j.smrv.2007.12.004 18313952

[19] Dell'osso B Lader M. Do benzodiazepines still deserve a major role in the treatment of psychiatric disorders? A critical reappraisal. Eur Psychiatry. (2013) 28:7–20. 10.1016/j.eurpsy.2011.11.003 22521806

[20] Irwin MR Valladares EM Motivala S Thayer JF Ehlers CL. Association between nocturnal vagal tone and sleep depth, sleep quality, and fatigue in alcohol dependence. Psychosom Med. (2006) 68:159–66. 10.1097/01.psy.0000195743.60952.00 16449427

[21] Crum RM Ford DE Storr CL Chan YF. Association of sleep disturbance with chronicity and remission of alcohol dependence: data from a population-based prospective study. Alcohol Clin Exp Res. (2004) 28:1533–40. 10.1097/01.ALC.0000141915.56236.40 15597086

[22] Brower KJ Krentzman A Robinson EA. Persistent insomnia, abstinence, and moderate drinking in alcohol-dependent individuals. Am J Addict. (2011) 20:435–40. 10.1111/j.1521-0391.2011.00152.x 21838842 PMC3156652

[23] Pengo MF Won CH Bourjeily G. Sleep in women across the life span. Chest. (2018) 154:196–206. 10.1016/j.chest.2018.04.005 29679598 PMC6045782

[24] Sierra JC Delgado-Dominguez C Carretero-Dios H. [Internal structure of dysfuncional beliefs and attitudes about sleep scale in a Spanish shift workers sample]. Rev Neurol. (2006) 43:454–60. 10.33588/rn.4308.2005733 17033977

[25] Bain KT. Management of chronic insomnia in elderly persons. Am J Geriatr Pharmacother. (2006) 4:168–92. 10.1016/j.amjopharm.2006.06.006 16860264

[26] Grau-López L Roncero C Grau-López L Daigre C Rodríguez-Cintas L Pallarés Y Factors related to relapse in substance-dependent patients in hospital detoxification: the relevance of insomnia. J Sleep Disor Treat Care. (2014) 3:3 10.1016/S0924-9338(14)78341-5

[27] Brower KJ Aldrich MS Robinson EA Zucker RA Greden JF. Insomnia, self-medication, and relapse to alcoholism. Am J Psychiatry. (2001) 158:399–404. 10.1176/appi.ajp.158.3.399 11229980 PMC3008542

[28] Grau-López L Daigre C Grau-López L Rodriguez-Cintas L Egido A Casas M. Administrative prevalence of insomnia and associated clinical features in patients with addiction during active substance use. Actas Esp Psiquiatr. (2016) 44:64–71. 27099212

[29] World Medical Association World medical association declaration of Helsinki. Ethical principles for medical research involving human subjects. Bull World Health Organ. (2001) 79:373–4. 11357217 PMC2566407

[30] Verkhratsky A Nedergaard M Steardo L Li B. Editorial: sleep and mood disorders. Front Psychiatry. (2020) 16:981. 10.3389/fpsyt.2019.00981 32010001 PMC6977539

[31] Steardo L de Filippis R Carbone EA Segura-Garcia C Verkhratsky A De Fazio P. Sleep disturbance in bipolar disorder: neuroglia and circadian rhythms. Front Psychiatry. (2019) 10:501. 10.3389/fpsyt.2019.00501 31379620 PMC6656854

[32] Faulkner S Sidey-Gibbons C. Use of the pittsburgh sleep quality index in people with schizophrenia spectrum disorders: a mixed methods study. Front Psychiatry. (2019) 10:284. 10.3389/fpsyt.2019.00284 31143131 PMC6520598

[33] Huang Z Zhan S Chen C Li N Ding Y Hou Y. The effect of insomnia on cortical excitability in patients with generalized anxiety disorder. Front Psychiatry. (2019) 9:755. 10.3389/fpsyt.2018.00755 30687140 PMC6335338

[34] Raffray T Bond TL Pelissolo A. Correlates of insomnia in patients with social phobia: role of depression and anxiety. Psychiatry Res. (2011) 189:315–7. 10.1016/j.psychres.2011.03.004 21463903

[35] Grau-López L Grau-López L Daigre C Palma-Álvarez RF Rodriguez-Cintas L Ros-Cucurull E. Pharmacological treatment of insomnia symptoms in individuals with substance use disorders in Spain: a quasi-experimental study. Subst Use Misuse. (2018) 53:1267–74. 10.1080/10826084.2017.1402056 29185897

[36] Daigre C Grau-López L Rodríguez-Cintas L Ros-Cucurull E Sorribes-Puertas M Esculies O. The role of dual diagnosis in health-related quality of life among treatment-seeking patients in Spain. Qual Life Res. (2017) 26:3201–9. 10.1007/s11136-017-1668-4 28786018

